# Lowland grazing and Marburg virus disease (MVD) outbreak in Kween district, Eastern Uganda

**DOI:** 10.1186/s12889-019-6477-y

**Published:** 2019-01-31

**Authors:** Aggrey Siya, William Bazeyo, Doreen Tuhebwe, Gabriel Tumwine, Arnold Ezama, Leonard Manirakiza, Donald R. Kugonza, Innocent B. Rwego

**Affiliations:** 1 College of Veterinary Medicine, Animal Resources and Biosecurity, Makerere University, P.O. Box 7062, Kampala, Uganda; 2 College of Health Sciences, Makerere University, P.O. Box 7062, Kampala, Uganda; 3 One Health Central and Eastern Africa, Kampala, Uganda; 4 Uganda Red Cross Society, P.O. Box 494, Kampala, Uganda; 5 The National Pharmacovigilance Center, National Drug Authority, P.O. Box 23096, Kampala, Uganda; 6 College of Agricultural and Environmental Sciences, Makerere University, P.O. Box 7062, Kampala, Uganda; 7 Department of Veterinary Population Medicine, College of Veterinary Medicine, University of Minnesota, St. Paul, MN USA

**Keywords:** Agriculture, Livelihoods, Zoonotic diseases, One health

## Abstract

**Background:**

Uganda is one of the few countries in Africa that has been experiencing outbreaks of viral hemorrhagic fevers such as Ebola, Marburg and Crimean-Congo Hemorrhagic fevers. In 2017 Uganda experienced a Marburg Virus Disease (MVD) outbreak with case fatality rate of 100% in Kween district. Although hunting for wild meat was linked to the MVD outbreak in Kween district, less was reported on the land use changes, especially the changing animal grazing practices in Kween district.

**Methods:**

Through Makerere University One Health graduate fellowship program with attachment to Uganda Red Cross Society, a study was conducted among the agricultural communities to elucidate the risk behaviors in Kween district that can be linked to the 2017 Marburg disease outbreak.

**Results:**

Results show that although a few elderly participants ascribed fatal causes (disobedience to gods, ancestors, and evil spirits) to the MVD outbreak during FGDs, majority of participants linked MVD to settling in caves (inhabited by Fruit Bats) during wet season as upper belts are extensively used for crop production leaving little space for animal grazing. Members also noted side activities like hunting for wild meat during this grazing period that could have predisposed them to Marburg Virus.

**Conclusions:**

There is need to integrate One Health concepts within agricultural extension service provision in Uganda so as to enhance the management of such infectious diseases.

## Background

Marburg Virus Disease (MVD) is one of the viral hemorrhagic fevers that affects both humans and non-human primates [7]. First outbreaks of Marburg occurred in Germany in 1967 with links to monkeys which were imported from Uganda for research purposes [5]. Marburg Virus Disease is caused by a filovirus similar to Ebola that belongs to the family *Filoviridae*. The Marburg virus causes severe viral hemorrhagic fever in humans with case fatality rates ranging between 24 and 88% [25]. *Rousettus aegypti* fruit bats of the Pteropodidae family, are considered to be natural hosts of Marburg virus [25]. The Marburg virus is transmitted to people from fruit bats and spreads among humans through human-to-human transmission [17]. Several outbreaks of hemorrhagic fevers have been recorded in Uganda with some linked to foreign travels to Uganda [14].

Four MVD outbreaks have been reported in Uganda [14, 18]. The first recorded outbreak was in 2007, where three cases and one death were reported in a community associated with mining activities in the districts of Kamwenge and Ibanda, Western Uganda [10]. In 2012, MVD was responsible for 26 cases with 15 deaths affecting multiple districts and in 2014, Kampala, Uganda’s capital city so far has reported only one case diagnosed with Marburg virus [18]. Outbreaks of MVD are believed to occur because of close interaction of humans and animals such as non-human primates, bats, and livestock [27]. Most of the Marburg disease outbreaks in Uganda have been linked to bat species *Rousettus aegypti* [10]. The most recent outbreak of Marburg in Kween district, Eastern part of Uganda in 2017 was linked to consumption of meat from a bat by a hunter [26]. This is similar to other outbreaks like the one in Ibanda and Kamwenge districts of Western parts of Uganda [1].

Apart from effects related to morbidity and mortality, the disease disrupts the country’s economy, causes social suffering of the affected families and reduces household productivities among others [27]. Its ability to spread beyond borders implies it can disrupt trade between countries reducing foreign exchange. The impacts of Marburg disease outbreaks are similar to the impacts of other hemorrhagic fevers like Ebola [11] that have also caused havoc in the Eastern Africa region in the recent past.

Most of these zoonotic disease outbreaks occur as a result of the changing economic activities that increase human-animal interactions [27]. Such changing economic activities include conversion of natural forests into farmland increasing chances of flooding which is a risk factor for diseases like leptospirosis [27]. Examination of a mine in northeastern Democratic Republic of Congo revealed bats that were found to have Marburg viruses on being examined [20]. This implies that the people who worked in such a mine risked contracting the Marburg viral disease. Uganda currently uses multidisciplinary approaches under the auspices of National One Health Task Force during investigation of such outbreaks. Makerere University is also embracing One Health approaches in community engagement through pairing up students with organizations like Uganda Red Cross Society practicing One Health approaches.

The 2017 MVD outbreak in Kween district was the first of its kind and was linked to hunting of bats. However, little has been done thus far to study the economic activities undertaken by the people of Kween that might predispose people to getting in contact with potential hosts for MVD. This research therefore focused on cattle rearing practices by the people of Kween, Eastern Uganda and their risk of contracting Marburg disease. This research focused on lowland grazing which normally happens during wet seasons of the year and corresponded with the 2017 Marburg disease outbreak.

## Methods

### Study area

**Fig. 1:**
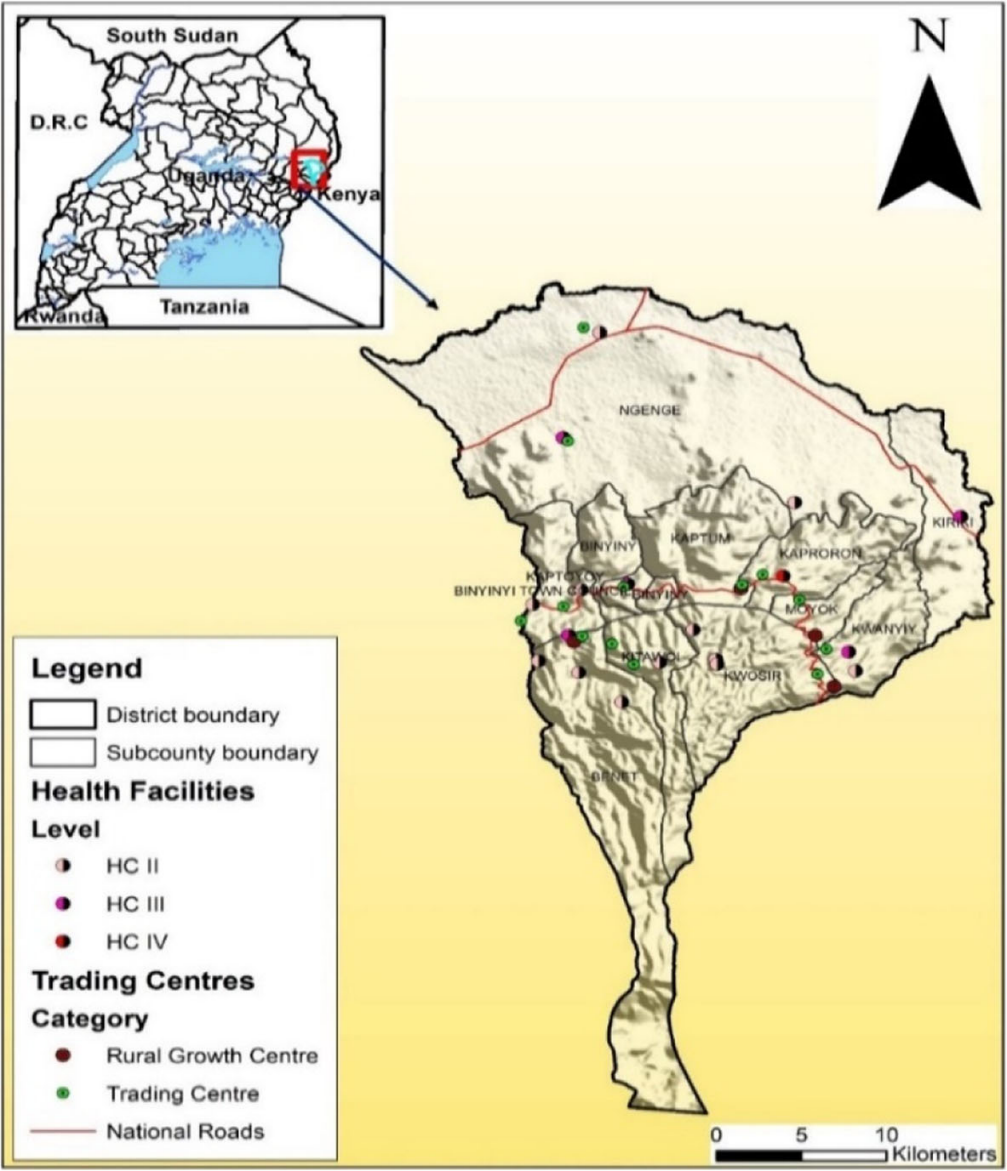
Location of the study area (Kween district)

The study was undertaken in Kween District in Eastern Uganda located at 01 25 N, 34 31E (Fig. 1). Kween district borders several districts namely: Nakapiripirit to the north, Amudat to the northeast, Bukwo to the east, Kapchorwa to the west and Bulambuli to the northwest [16]. In the south, it boarders with the Republic of Kenya. The district headquarters are located at Binyiny; approximately 69 km by road from Mbale, one of the largest towns in eastern Uganda. Kween district is one of the youngest districts in Uganda having been created by the Act of Parliament and becoming operational on 1st July 2010. Originally, it was part of Kapchorwa district and together with Kapchorwa and Bukwo district, they form the Sebei sub-region. The district is located on the northern slopes of Mount Elgon, at an average altitude of about 1900 m (6,200 Feet) above sea level.

In the three-national population census especially of 1991, 2002 and 2012, the district population was estimated at 37,300, 67,200 and 103,300 respectively Uganda Bureau of Statistics [23]. Its population growth rate of averagely 4.5% is above the national average of 3.6% [22]. The major economic activity is farming and the major crops grown are; maize, beans, bananas, wheat, barley and cowpeas and livestock rearing like chicken, goats, cattle among others [21]. The district is located on the slopes of Mt. Elgon characterized by high and well distributed rainfall (averaging 1200 mm/year) and is characterized by two seasons, a rainy season (March–September) and a dry season (October–April) [13]. It has cool temperatures (averaging 17 °C), its soils are predominantly volcanic [3].

### Study setting and participants

This study was conducted following the One Health fellowship attachment to Uganda Red Cross Society Community Pandemics Preparedness Project (CP3). Uganda Red Cross Society being one of the organizations practicing One Health approaches was identified by Makerere University One Health Institute for the graduate fellowship attachment. The Uganda Red Cross Society identified their branch in Kapchorwa as one of the areas a fellow can gain experience on the need for multidisciplinary teams to address disease outbreaks. A wide range of stakeholders within the district were involved in the study including local council leaders, officials from department of production, environmental officers and community development officers.

### Data collection

This study utilized a cross sectional study design [6] in 2018 using a semi-structured questionnaire [15] during focus group discussions.

### Focus group discussions

The study used data generated from focus group discussions (FGDs) undertaken in Kaptum and Kaproron sub counties, both located in the mid-latitudes of the Kween district, Uganda and within the lowland areas where livestock grazing is predominant during rainy season. The two communities were selected because they are near the lowlands and also represent the communities living up-high on the slopes of Mount Elgon within the district. About six (6) Focus Group Discussions (FGDs) were conducted in the two study communities. About three FGDs were undertaken in each of the two communities. Sixty (60) participants nominated by community members to represent them took part in the FGDs. FGD participants were grouped according to age and gender. Both male and female adults were aged 25 years and above.

The participants were also classified based on their education status and sources of income. The challenges faced by the participants in pursuing their livelihoods were noted during the discussion process. Pre-designed focus group discussion guide were used and the discussions were conducted. A facilitator, note-taker and a tape recorder were utilized during the discussions. The discussions revolved around perceptions of participants on the link between lowland grazing and MVD and on the cattle grazing practices undertaken in the lower belt. The FGDs were conducted in *Kupsabiny*, the local language mostly used in the area. Consent was obtained from the participants and community leaders before the study.

### Data analysis

For qualitative data from FGDs, analysis was conducted using thematic framework [24]. This involved; familiarization, and identification of thematic frameworks; indexing; charting; mapping and interpretation. Recorded audio tape was listened to and the transcripts and observational notes taken during the discussions were used to enhance familiarization with the information gathered. This allowed identification of emerging dominant themes during the discussions. In indexing, analysis of the transcript was undertaken with use of codes from the responses obtained in order to sort out interesting quotes from the transcripts with the aid of NVivO coding [9]. A determination was then made of the emerging themes in line with the study objectives. Subsequently, a coding frame was developed and coded data was mapped and interpreted. Throughout this, particular attention was paid to the context, comments, and words in order to enhance the internal consistency of the data. Triangulation techniques, in the form of discussions with key community leaders were used to reduce potential bias effects in the data.

## Results

### Demographics of participants

Around 60 respondents were interviewed during this study with ages of about 51 ± 24 years. Out of these respondents interviewed from Kaptum and Kaproron, more than half (55%) of them were from Kaptum Sub County while the rest (45%) came from Kaproron Sub County (Table 1). About three quarters (73%) of the total respondents were male. In terms of education status, only 36% of them had attained at least secondary level education implying the limited capacity of human capital in such societies to tap into a variety of livelihood options to enhance their resilience to shocks.

**Table 1:** Summary of socio-demographic characteristics of respondents

Characteristic	Level	Frequency (*n* = 60)	Percentage
Sub County	Kaproron	27	45
	Kaptum	33	55
Gender	Male	44	73
	Female	16	27
Education level	No Education	12	20
	Primary	26	43
	Secondary	11	18
	Diploma/Certificate	6	10
	Post graduate	2	3
	Unknown	3	5
Marital status	Married	50	83
	Widowed	6	10
	Divorced	1	2
	Undisclosed	3	5

### Land ownership and economic activities in Kween district

The average land owned by participants was 11.5 ± 16.8 acres per person (Table 2). All the respondents that were interviewed owned land with majority (90%) on free hold tenure. Also, majority (98%) of the respondents had documents including purchase agreements (82%) as a proof of land ownership. The main economic activities carried out by the respondents were crop growing and livestock farming (87%) yielding an average income of 3.3 million Uganda shillings (Table 2).

**Table 2:** Land ownership in Kween district

Variable	Level	Frequency	Percentage
Land ownership	Yes	60	100
	No	0	0
Land tenure system	Communal	1	2
	Freehold	54	90
	Leasehold	5	8
Document ownership	Yes	59	98
	No	1	2
Type of document	Purchase agreement	49	82
	Written Will	10	17
	None	1	2
Average land size (acres)		11.5 ± 16.8	
Average income (UgX		3,353,208 ± 5,542,505.95	

More than three quarters of the land (90%) was under free hold tenure system allowing for opportunities to ensure sustainable land management that can ensure reduced outbreak of infectious diseases like Marburg due to reduced human-animal interaction.

### Challenges faced in undertaking various economic activities in Kween district

The greatest challenges faced by farmers were pests and diseases (40.7%) followed by drought (23.7%). Other challenges faced include dry spell in the district, high mortality rates in animals and lack of resources (Table 3).

**Table 3:** Challenges faced by cattle keepers in Kween district, Uganda

Challenges faced	Frequency	Percentage
Pests & diseases	24	40.7
Dry spell	14	23.7
High mortality rate of animals	4	6.8
Theft	3	5.1
Limited funds	2	3.4
Limited land	2	3.4
Limited Market	2	3.4
Others	8	13.6

Most of the farmers (43.3%) were spraying using pesticides to curb the challenge of pests and diseases in the area. In addition, 21.7% were practicing irrigation to solve the challenge of drought, while 10% had planted resistant crop varieties to curb the challenge of crop diseases. The rest practiced contract farming and early farming (3.3% each) and other solutions to help many the challenges they were facing. While they practiced the above to solve the challenges they were facing, they mentioned other opportunities within the district that allow mitigation of the challenges the challenges. The respondents mentioned that the main opportunities available to solve the challenges were availability of agrochemicals, improved seed varieties and availability of financial services.

### Activities undertaken by farmers and their link to Marburg outbreak

i. Hunting

Hunting was one of activities carried out in the study area. Respondents explained that in most times hunting was done while grazing to get food to eat since there are no crops;

*“Since there are no crops, people leave their animals to freely graze and go for activities like hunting.”* FGD member

*“The men set simple houses and some of them during grazing go for hunting because so many wild animals are there.”* Another member explained

The respondents emphasized that some people hunt for monkeys and baboons and even eat them which could be one of the risk factors for Marburg outbreak in Kween district;

*“On the slopes, near caves, the baboons and monkeys destroy crops and people sometimes trap them alongside animal rearing in lowland areas. They can even eat them”.* One respondent answered

One respondents explained the perceptions of these people who kill and eat monkeys, that the monkey’s liver is medicinal;

*“Some people even kill these monkeys and I heard that there are some people who eat them. They say its liver is medicinal.”* FGD member

*“Some boys kill animals like baboons which are not edible. They just do it for fun. Some of them even hunt for birds and roast them after killing.”* FGD member

Some respondents also explained that the activities in lowland could be risk factors for Marburg outbreak, especially cleaning of caves that are inhibited by bats;

*“Let me add on the issue of Marburg, I think it’s because of our activities in the lowland areas. We kill so many animals during our free time. Some people also clean the caves which are inhabited by bats and risk contracting this strange disease”.* FGD member

ii. Grazing

Most of the people in Kween district practice livestock farming as one of their economic activities. They said that during grazing, they set up simple houses and kraals for them and their animals to stay in;

*“We set up kraals and construct simple houses. The goats stay in caves.”* FGD member

*“Construction of simple houses occurs which eventually becomes rooms for human settlement while cattle stay in simple kraals. The goats stay in caves.”* FGD member

*“The men set up simple houses and in most cases cook for themselves but when they come home, the women cook for them.”* FGD member

One respondent illustrated that there is peace in the lowlands for grazing, however, he said the bats which settle in the caves where their animals stay can cause Marburg and kill the whole family;

“*There used to be too much insecurity in the lowland areas because of the Karimojong cattle rustlers but now it’s peaceful and most of us take our animals there. However, there is a problem of Marburg now because of the bats which settle in caves and the disease they cause is very bad. It can kill the whole family.”* FGD member

They also said that the caves are accessed by wild animals such as monkeys and baboons which can cause Marburg;

*“Wild animals like monkeys, baboons among others have access to these caves and I think can cause Marburg.”* FGD member

The respondents also expressed their worries about staying near the caves due to fear of Marburg that had killed people in the area;

*“I’m now worried about staying in or near caves because there is a new disease called Marburg which killed some people this year.”* FGD member

iii. Mining

Another activity carried out in this area is salt mining that is done in caves. Respondents illustrated that they mine salt in caves and give to their animals to make them healthy. However, it was earlier explained how these caves are accessed by bats, monkeys and baboons that could cause Marburg;

*“The rock salt is mined and is very good for our animals. We get this from the cave and it makes the animal very healthy.”*

## Discussion

This is the first study conducted in Kween District looking at farmers perspectives around Marburg disease. The main challenges faced by farmers are pests and diseases. Even though grazing is one of the main activities, they did mention that hunting was common activity during grazing of animals. Setting of traps and eating of monkey meat was one of the activities identified in lowland areas during this study. Studies by Blumberg [4] among others have shown a link between eating bush meat and occurrence of viral hemorrhagic fevers such as Ebola. Results of this study show that community members have their own perspectives about Marburg like causes of its occurrence. This is similar to what Hewlett and Amolat [12] found out in Gulu district of Uganda following an Ebola outbreak. Local views about hemorrhagic fevers exists in the affected communities regarding its causes and control. Such local views about an infectious disease like Marburg can provide a measure of the level of knowledge within a community about filoviruses for proper design of public policy. A clear understanding of activities that can lead to Marburg Virus outbreak as illustrated in this paper allows for design of messages to ensure reduced risk of future outbreaks. Such issues and activities include; shortage of fertilizers for use in crop production resulting into use of droppings from bats, mining of rock salt in caves for animals among others. This is similar to the results of the 2012 Marburg disease outbreaks in Uganda in the districts of Ibanda [10]. However, studies by Green [10] and Adjemian et al. [1]did not consider other factors that drive people to doing the mining activities as highlighted in this study.

Farmers had a belief that bats can potentially spread Marburg to them and their animals. They also believe that access to caves and grazing lands by baboons and monkeys can potentially spread Marburg to farmers. This study is similar to that of Brauburger et al. [5] that linked the 1967 Marburg outbreaks in Germany to the monkeys that were imported from Uganda. However, his studies did not also include the community’s perspective about such disease outbreaks. Chandler et al. [8] also notes that design of messages of hope are key in containing infectious diseases like Marburg. A clear understanding of the economic activities like animal grazing are key in design and ensuring effectiveness of such messages and public policy. Natural resource management systems like land tenure systems are key in addressing infectious diseases like Marburg. According to Andersen [2], land tenure management systems are key to sustainable land management systems. Communal land ownership leads to a tragedy of commons where no one takes the responsibility of say clearing off growing *Lantana camara* that would provide conditions for settlement of wild animals that might be carrying disease causing pathogens. In this case, establishment of routes of escape and places of settlement for organisms harboring fruit bats and monkeys within human settlements can pose a risk of spillover of such pathogens like Marburg Virus to humans. Land tenure systems are therefore key in designing public policy to prevent emergence and reemergence of infectious diseases like Marburg disease. As O’Shea et al. [19] notes the likelihood of filoviruses evolving in response to changing environmental conditions due to bat migrations, in this case, settling in caves (habitats for fruit bats) during animal rearing in lowland areas can lead to migration of bats to more open habitats. Such movements can alter the metabolism of the bat causing alteration of the filoviruses like that of Marburg disease increasing its spill over to humans.

This study was however limited by the inability to interview individuals directly affected by Marburg disease in Kween district. The study was also conducted at a much later time when people were not remembering well information regarding Marburg disease.

## Conclusions

It was generally perceived that settling in caves during lowland grazing could increase the chances of contact with bats. Furthermore, the activities listed by participants, such as mining, grazing of cattle and hunting which are undertaken in the lowland areas can increase It was generally perceived that settling in caves during lowland grazing could increase the chances of contact with bats. Furthermore, the activities listed by participants, such as mining, grazing of cattle and hunting which are undertaken in the lowland areas can increase human-animal animal interaction. Although a few participants ascribed fatal causes (disobedience to gods, ancestors, and evil spirits) to the MVD outbreak during FGDs, majority of participants rightly linked MVD to settling in caves (inhabited by Fruit Bats) during wet season as upper belts are extensively used for crop production leaving little space for animal grazing. Members also noted side activities like hunting for wild meat during this grazing period that could have predisposed them to Marburg Virus. They were therefore of the view that future changes in animal rearing practices will further exacerbate the zoonotic diseases outbreaks in their communities if appropriate interventions are not put in place.

There is need to integrate One Health concepts into agricultural extension service provision in Uganda so as to enhance the management of such infectious diseases. All stakeholders involved in agricultural production (crop, and livestock) need to be acquainted with the concepts of One Health so as to ensure the environment-human-animal interaction is balanced. Otherwise leaving public health officers only to tackle such diseases wont yield much. There is need for more research also on the link between livelihood strategies like lowland grazing among others and Marburg disease outbreaks.

## Abbreviation

FGDS: Focus group discussions
